# Progressive drought alters the root exudate metabolome and differentially activates metabolic pathways in cotton (*Gossypium hirsutum*)

**DOI:** 10.3389/fpls.2023.1244591

**Published:** 2023-08-30

**Authors:** Heng-An Lin, Harrison R. Coker, Julie A. Howe, Malak M. Tfaily, Elek M. Nagy, Sanjay Antony-Babu, Steve Hague, A. Peyton Smith

**Affiliations:** ^1^Department of Soil and Crop Sciences, Texas A&M University and Texas A&M AgriLife Research, College Station, TX, United States; ^2^Department of Environmental Science, University of Arizona, Tucson, AZ, United States; ^3^Department of Plant Pathology and Microbiology, Texas A&M University and Texas A&M AgriLife Research, College Station, TX, United States

**Keywords:** root exudates, untargeted metabolomics, drought, FT-ICR MS, upland cotton, nondestructive sampling, aeroponics

## Abstract

Root exudates comprise various primary and secondary metabolites that are responsive to plant stressors, including drought. As increasing drought episodes are predicted with climate change, identifying shifts in the metabolome profile of drought-induced root exudation is necessary to understand the molecular interactions that govern the relationships between plants, microbiomes, and the environment, which will ultimately aid in developing strategies for sustainable agriculture management. This study utilized an aeroponic system to simulate progressive drought and recovery while non-destructively collecting cotton (*Gossypium hirsutum*) root exudates. The molecular composition of the collected root exudates was characterized by untargeted metabolomics using Fourier-Transform Ion Cyclotron Resonance Mass Spectrometry (FT-ICR MS) and mapped to the Kyoto Encyclopedia of Genes and Genomes (KEGG) databases. Over 700 unique drought-induced metabolites were identified throughout the water-deficit phase. Potential KEGG pathways and KEGG modules associated with the biosynthesis of flavonoid compounds, plant hormones (abscisic acid and jasmonic acid), and other secondary metabolites were highly induced under severe drought, but not at the wilting point. Additionally, the associated precursors of these metabolites, such as amino acids (phenylalanine and tyrosine), phenylpropanoids, and carotenoids, were also mapped. The potential biochemical transformations were further calculated using the data generated by FT-ICR MS. Under severe drought stress, the highest number of potential biochemical transformations, including methylation, ethyl addition, and oxidation/hydroxylation, were identified, many of which are known reactions in some of the mapped pathways. With the application of FT-ICR MS, we revealed the dynamics of drought-induced secondary metabolites in root exudates in response to drought, providing valuable information for drought-tolerance strategies in cotton.

## 1 Introduction

Root systems release a large variety of compounds into the soil environment, commonly referred to as root exudates (Badri and Vivanco, 2009). Root exudates are mainly comprised of various primary and secondary metabolites, such as amino acids, sugars, phenolics, and plant hormones (Oburger and Jones, 2018). Root exudation is an important process that regulates interactions between plants, soil, and soil microorganisms in response to environmental stimuli (Bais et al., 2006; Baetz and Martinoia, 2014; Canarini et al., 2019). Plants may strategically modify the quantity and quality of their exudate profile in response to different abiotic and biotic stresses (Carvalhais et al., 2011; Gao et al., 2018; Li et al., 2019; Zhang et al., 2020), shaping the composition and activity of the rhizosphere microbiome, and promoting ecological feedback to plant hosts. For example, increased release of *γ*-aminobutyric acid (GABA) and carbohydrates was observed in the root exudates of P-deficient maize (Carvalhais et al., 2011). GABA has been known to regulate anion channels associated with malate efflux (Ramesh et al., 2015), and malate is one of the critical compounds that soybean (*Glycine max*) exudes to mobilize inorganic P in soil (Krishnapriya and Pandey, 2016). The release of carbohydrates into the rhizosphere has also been linked to enhancing germination and colonization of mycorrhizal fungi, which could improve P acquisition (Bücking et al., 2008; Etesami et al., 2021). The role of root exudates in inducing plant defense response or suppressing root diseases has also been reported in different hosts, such as wheat (Li et al., 2019), soybean (Gao et al., 2018), and tobacco (Zhang et al., 2020). While interest in root exudate research has been primarily focused on plant nutrient acquisition and the signal transductions between plant host and rhizosphere microbiome, there has been recent attention towards the effect of drought on root exudates, rhizosphere microbiomes under drought conditions, and these effects on ecosystem-scale responses (e.g., carbon sequestration, soil respiration, soil aggregation, soil organic matter decomposition, and plant-microbe symbiotic relationships) (Preece and Peñuelas, 2016; Karlowsky et al., 2018; de Vries et al., 2019; Williams and de Vries, 2020).

Several characteristics of root exudates undergo changes during drought. For example, drought-resistant maize (*Zea mays*) varieties exuded 58.2% more mucilage compared to drought-susceptible varieties (Nazari et al., 2023), and the role of mucilage has been linked to enhanced hydrologic conductance at the root-soil interface (Ahmed et al., 2014). The amino acid proline, an osmotic regulator, is known to accumulate in root exudates under drought in citrus (*Citrus* sp.) (Vives-Peris et al., 2017) and during recovery in pea (*Pisum sativum*) (Rubia et al., 2020). However, previous works have shown a range of responses in exudation rate and quantity of targeted compounds in response to drought. For example, increased exudation rates of carbon under drought have been reported in wheatgrass (*Agropyron cristatum*), mountain grasses, sunflower (*Helianthus annuus*), and holm oak (*Quercus ilex*) but not soybean (*Glycine max*) (Henry et al., 2007; Canarini et al., 2016; Karlowsky et al., 2018; Preece et al., 2018). Moreover, the variability of the drought effect on root exudates or the co-occurrence of multiple stressors [e.g., heat and drought stress (Tiziani et al., 2022)] presents a challenge in predicting how a particular plant species will respond to drought, as plant-soil interactions of different crops lack clear patterns associated with drought treatment or duration (Preece and Peñuelas, 2016). While a recent study hypothesized that root exudation patterns are linked to plant growth strategies and align with the ecosystem response to drought (Williams and de Vries, 2020), the use of various and inconsistent approaches in root exudate collection, different durations and severities of drought treatments, and varying analytical methods have made it challenging to compare the literature and draw conclusive results. In addition, the genetic effects (i.e., species, cultivars), plant growth stage effects, and other environmental factors (e.g., temperature, nutrient status, wind and light) have further complicated the understanding of the relationship between root exudation and drought response. Therefore, there is a need to reveal drought-induced exudation profiles for major crops with advanced technologies to identify potential drought-tolerance regulators.

Analytical techniques such as gas chromatography coupled to mass spectrometry (GC-MS), liquid chromatography-MS (LC-MS), and/or nuclear magnetic resonance spectroscopy (NMR) are often used to characterize the metabolome of plant root exudates (Fan et al., 2001; Luo et al., 2017; Gargallo-Garriga et al., 2018; Salem et al., 2022). Fourier-transform ion cyclotron resonance-MS (FT-ICR MS) is an ultrahigh-resolution analytical technique that has also been proven as a powerful tool for qualitative characterization of the metabolome of complex plant extracts (Maia et al., 2021) and for characterizing the root exudate composition of different plant species (Miao et al., 2020; Lohse et al., 2022; Ulrich et al., 2022). A shift of root exudate’s metabolome profile under different levels of drought has been identified in holm oak (*Quercus ilex*) using LC-MS (Gargallo-Garriga et al., 2018) and blue grama (*Bouteloua gracilis*) using GC-MS, NMR, and FT-ICR MS (Ulrich et al., 2022). Both studies demonstrated the dynamics of root exudate compositions and how they corresponded with the severity of drought. While the choice of analytical technology depends on multiple factors, such as high throughput capacity, optimal metabolome coverage, and other factors, FT-ICR MS yields high mass accuracy and resolution power, with a mass-to-charge ratio (m/z) range of 200 to 900 (Hsu et al., 2011; Park et al., 2013), which offers valuable insight into elemental and compound diversity of plant secondary metabolites (Takahashi et al., 2008; Maia et al., 2021). Further, by mapping the molecular formula to databases reveal potential functional pathways and direct the discovery of novel compounds as inherent advantages that are not as achievable with traditional approaches (Salem et al., 2022).

Drought has severe effects on cotton production. For example, in the Southwest region of the United States, a record crop abandonment rate 71% occurred in 2022 due to extreme and unexpected drought throughout the planting and growing season (Meyer et al., 2023). Compared with the previous season’s 12% abandonment rate, this led to a 40% decrease in cotton production in 2022 (Meyer et al., 2023). Therefore, disentangling the molecular feedback between root exudates and plant hosts in response to drought is crucial, as drought is predicted to increase in frequency due to climate change (IPCC, 2021). In this study, we deciphered the qualitative molecular characteristics of root exudates in upland cotton (*Gossypium hirsutum*), an important fiber crop commonly grown in water-restricted areas. We hypothesized the exudation profile would shift under progressive drought stress. More specifically, unique metabolites would be released during drought, which might be involved in plant stress response or act as signal molecules to interact with beneficial microbes for stress resilience. The objectives were to (1) characterize organic compounds in cotton root exudates under progressive drought stress using untargeted ultra-high resolution mass spectrometric technology (FT-ICR MS), (2) identify key metabolic pathways induced by drought, and (3) identify metabolic profile shifts throughout intensifying drought stress.

## 2 Materials and methods

### 2.1 Experimental design

**Figure 1:**
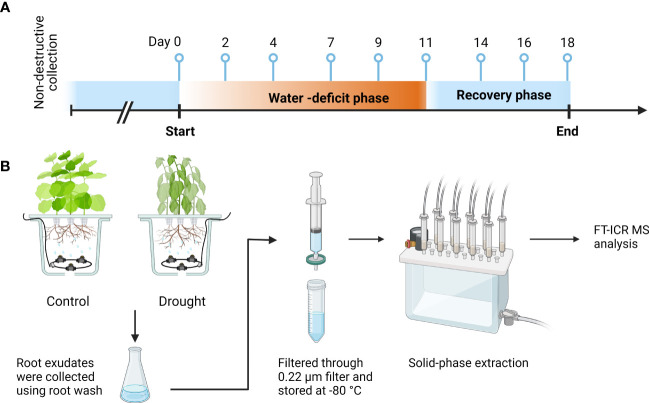
Sample collection timeline and sample processing. **(A)** Root exudates were collected at 0, 2, 4, 7, 9, 11, 14, 16, and 18 days after the experiment was initiated, which includes a baseline (day 0), water-deficit phase (day 2, 4, 7, 9, and 11), and recovery phase (day 14, 16, and 18). **(B)** Root exudates collected from drought-treated and control-treated plants were filtered through 0.22-μm syringe filter, desalted, and concentrated by solid phase extraction, and analyzed with FT-ICR MS. Each treatment consisted of five pots with three plants per pot. This figure was created with BioRender.com.

An aeroponic growth chamber trial was performed in 2021. The pots were arranged in a completely randomized design with two treatments, progressive drought stress (drought) and well-watered control (control). Each treatment consisted of 10 pots and each pot had three plants. Treatments were applied for 11 days (water-deficit phase) once plants reached the match-head square stage (Elsner et al., 1979). We specifically targeted this growth stage for cotton because it is particularly vulnerable to drought, which can result in a severe reduction in yield and fiber quality (Zonta et al., 2017). The control treatment received full irrigation (130 mL hr^-1^)of full-strength Hoagland solution (Hoagland and Arnon, 1950) throughout the entire experiment, while the drought treatment received a 50% reduction in irrigation every 2 to 3 days that ended with 10% full irrigation (water-deficit phase). After 11 days of treatment, irrigation was returned to 100% for all pots for 7 days (recovery phase). Root exudates were non-destructively collected at 0, 2, 4, 7, 9, 11, 14, 16, and 18 days after the experiment was initiated, which includes a baseline (day 0), water-deficit phase (day 2, 4, 7, 9, and 11), and recovery phase (day 14, 16, and 18). Five out of ten pots were randomly selected for sample collection on each sampling day. A total of 90 samples were collected, which consisted of 2 treatments and 5 replicates for 9 sampling days (**Figure 1**).

### 2.2 Plant materials, hydroponic, and aeroponic systems

A moderately drought susceptible upland cotton (*Gossypium hirsutum*, cv. TAM 421) was chosen for this study. Cotton seeds were surface sterilized with 1 min of 70% EtOH, 3 min of 2% NaOCl, and rinsed with sterile distilled water 3 times. After the seeds germinated on the germination papers, three seedlings were placed in individual mesh net cups and supported with hydroponic sponges in each pot (~3.8 L). The hydroponic system was set up with an air pump equipped with two 10-way splitters. Each valve on splitter was connected to an air stone with airline tubing for each pot. Nutrients were supplied with a full-strength Hoagland solution (Hoagland and Arnon, 1950), where the nutrient solution was replaced twice a week, maintaining a pH range of 6.0-6.5. Plants were grown in a hydroponic system with constant aeration with dissolved oxygen levels ranging between 7.7 and 8.0 mg L^-1^ until 3 to 4 true leaves had developed. Then, the plants were transferred to an aeroponic system with 3 plants per pot. The set up of aeroponic system and the misting cycle was followed by Lin et al. (2022, *preprint*). In brief, the nutrient solution was pressurized using a diaphragm pump with a built-in pressure shut-off switch set to 827.3 kpa (120 psi). The pressurized solution was then temporarily stored in a 0.75 L pressure accumulator tank before it was delivered to each pot. The misting cycle was controlled by digital timers. For each pot (~7.6 L) in the aeroponic system, a spray ring was installed at the bottom (**Figure 1B**), composed of 3 nozzles (0.1 mm diameter). Misting was set for 10 sec, and time between misting events ranged between 5 min (well-watered) and 50 min (extreme drought). Environmental conditions in the walk-in growth chamber (EGC, USA) were set as 12-hour light/12-hour dark photoperiod. The air temperature and relative humidity settings were based on the local weather station to mimic the field environment (**Supplementary Table 1**). A complete description of plant growth conditions, aeroponic system design, and plant performance were reported in Lin et al. (2022, preprint). In brief, the drought symptoms (less than 5% of leaf affected) were first visually observed on day 4 of the drought treatment. The symptoms reached the greatest severity (over 66% of leaf affected) on day 9 and were approaching the wilting point on day 11. The plants in the drought treatment were visually fully recovered by the end of recovery phase.

### 2.3 Root exudate collection

Root exudates were collected from the whole root system in the aeroponic system at 14:00-16:00 on each collection day (Lin et al., 2022, preprint). The choice of the sampling time was based on our preliminary studies, which revealed a trend of higher concentrations of a targeted compound, abscisic acid, during the afternoon collections. In brief, roots were rinsed with a sampling solution (0.05 mM CaCl_2_, pH 6.0-6.5) for 2 min using the aeroponic system with a clean mist collection container. The choice of a dilute CaCl_2_ solution was designed to reduce the drastic change in ionic strength and mimic the soil solution, which is typically dominated by Ca^2+^ ions. The collected exudates were then filtered through a 22-μm polyethersulfone syringe filter and aliquoted into 1.5 mL centrifuge tubes. Samples were then stored at -80°C until further analysis.

### 2.4 Sample preparation

The samples were desalted and concentrated by solid phase extraction (SPE) using the method by Dittmar et al. (2008). The SPE cartridge (Bond Elut PPL, 100 mg, Agilent, CA, USA) was rinsed with 3 mL of methanol (one cartridge volume) to activate the column. A 1 mL aliquot of root exudates was first diluted with 14 mL of sterile double-distilled water and acidified with 1 M HCl until the pH was between 2 to 3. The samples were then vacuumed through the SPE cartridge under 170 mbar. Prior to elution, a total of 30 mL of 0.01 M HCl was rinsed through the cartridge to remove possible salt molecules and interferents. After allowing the sorbents to air dry, samples were eluted with 1.5 mL of methanol. Extracted samples were stored at -20°C until analysis.

### 2.5 Fourier transform ion cyclotron resonance mass spectrometry (FT-ICR MS)

Extracted samples were analyzed with a 9.4 Tesla Bruker FT-ICR spectrometer located at the University of Arizona. A standard Bruker electrospray ionization (ESI) source was used to generate negatively charged molecular ions, where samples were introduced *via* direct infusion to the ESI source. 144 scans were averaged for each sample and internally calibrated using an organic matter homologous series separated by 14 Da (CH2 groups). Data Analysis software (BrukerDaltonik version 4.2) was used to convert raw spectra to a list of m/z values applying FTMS peak picker module with a signal-to-noise ratio (S/N) threshold set to 7 and absolute intensity threshold to the default value of 100. Putative chemical formulae were then assigned using Formularity (Tolić et al., 2017) software as previously described in Tfaily et al. (2018). Chemical formulae were assigned based on the following criteria: S/N > 7 and mass measurement error < 1 ppm, taking into consideration the presence of C, H, O, N, S and P and excluding other elements. The data produced by FT-ICR MS (peak masses, peak intensities, and metabolic molecular formula) were then processed through Metabodirect pipeline (Ayala-Ortiz et al., 2023). Peak masses (m/z) < 200 and > 900 were filtered using -m option to target secondary metabolites (Hsu et al., 2011; Park et al., 2013). Data was z-score normalized using –norm_method option. The quality control steps, including 13C isotope filtering and error filtering (0.5 ppm), followed the default setting. Masses must be identified in at least two samples to be included in the analysis. Elemental types (CHO, CHON, CHONP, CHONS, CHONSP, CHOP, CHOS, and CHOSP) were assigned for the filtered masses. KEGG analysis and mass difference analysis were performed using -k and -t options, respectively. Mass differences were used to infer potential biochemical transformations. Potential functional characteristics of the assigned molecular formulas were obtained by mapping to the KEGG database. Despite the molecular formula being the only criterion used for mapping to KEGG, it is important to acknowledge that these assignments are tentative. As noted, there is the possibility of multiple KEGG metabolites, particularly isomers, having the same molecular formula. Therefore, the molecular formula assignments should be interpreted with caution. The drought-induced unique metabolites in water-deficit phase (day 2 to 11) were characterized by comparing with control-treated samples excluding the drought-unique peaks identified at baseline (day 0). The KEGG pathways that were associated with human metabolisms were manually removed from the annotation list to generate the count table. The number of drought-unique metabolites were further visualized with UpSet plot (Lex et al., 2014) to identify the intersections between days (day 2 to 11). The figures were generated with R 4.2.0 (R core team, 2021) or GraphPad Prism v.6.0 (GraphPad Software, La Jolla, CA).

### 2.6 Statistical analysis for elemental compositions

The abundance matrix of elemental compositions was used to compute Euclidean distance matrices using vegdist function in R package vegan (Oksanen et al., 2020). The data were subset by experimental phases (i.e., baseline, water-deficit, and recovery) to generate each distance matrix. Permutational multivariate analysis of variance (PERMANOVA) analysis was performed for each experimental phase to assess the experiment effect using the adonis2 function in R package vegan (Oksanen et al., 2020). In a PERMANOVA model, the response variable was the distance matrix, and the explanatory variables were treatment (drought and control), days of treatment, and the interaction of treatment × days. Principal component analysis (PCA) was performed to detect the patterns in the elemental compositions using prcomp function in R package stats (R core team, 2021).

## 3 Results

### 3.1 Drought-induced unique metabolites were identified in cotton root exudates during water-deficit phase

**Figure 2:**
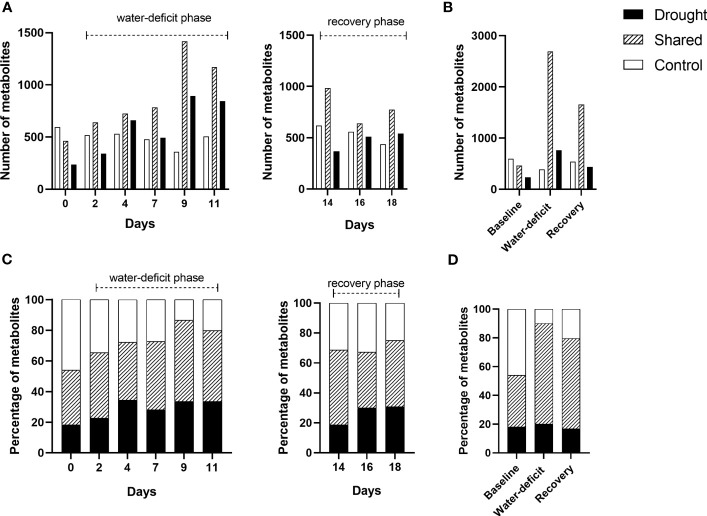
Stacked bar chart showing the number and percentage of shared and unique metabolites between control and drought treatments (n=5) throughout baseline (day 0), water-deficit (day 2-11) and recovery phases (day 14-18). Number of metabolites (assigned molecular formula) by day **(A)** and phase **(B)**. Percentage of metabolites by day **(C)** and phase **(D)**.

A total number of 33,870 m/z were identified by FT-ICR MS. After the data was filtered using quality control steps (Ayala-Ortiz et al., 2023), a total of 13,033 m/z remained. After formula assignment, there were 3,985 metabolites with an assigned molecular formula. Overall, the total number of metabolites increased by 78% as drought stress progressed from day 2 to 11 (**Figure 2**). The highest number of total metabolites and drought-unique metabolites were identified under severe drought (day 9), the time point prior to wilting point (day 11) (**Figure 2**). Up to 70% of metabolites were shared between drought and control treated plant during water-deficit phase (**Figure 2D**). During the water-deficit phase, there were two discrete sampling days when the total number of metabolites increased (**Figures 2A, C**). The first increase occurred on day 4 of the drought treatment when the initial drought symptoms visually appeared. The second increase was observed on day 9, when the plants reached severe drought. Under drought, there was a reduction of the total number of metabolites at day 11 (i.e., near permanent wilting point) and a decrease throughout the recovery phase (day 14-18) (**Figures 2A, B**). The described trend in number of metabolites corresponded to the drought-unique metabolites. When grouping the data by phase, a greater total number of metabolites and drought-unique metabolites were observed in the water-deficit phase compared to day 0 and recovery phase (**Figures 2B, D**). Unique metabolites at baseline were identified between drought and control samples and those metabolites were excluded in some of the following analysis (**Figure 2**).

### 3.2 Elemental composition in cotton root exudates

**Figure 3:**
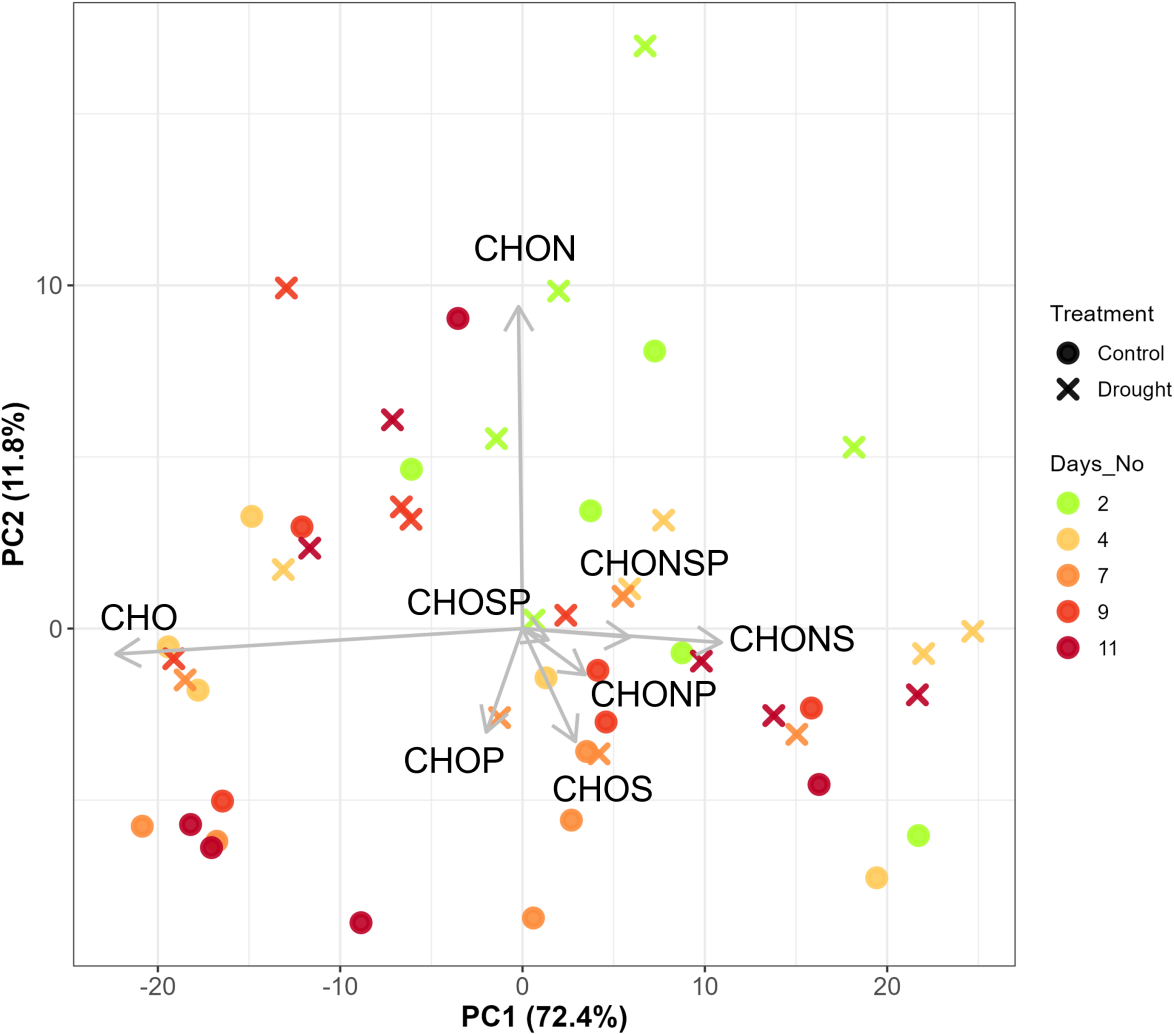
Principal Component Analysis (PCA) biplot showing the variation elemental compositions of root exudates during water-deficit phase (day 2-11, n=5). The arrows were labeled by the elemental types.

PERMANOVA showed no differences between treatment, days, and treatment × days interaction in elemental composition (i.e., CHO, CHON, CHONP, CHONS, CHONSP, CHOP, CHOS, and CHOSP) when all compounds were used. Treatment had a nearly significant effect on elemental composition (*p*-value=0.07) during the water-deficit phase (**Table 1**). PCA analysis of elemental composition showed a minor effect of treatment during water-deficit phase (**Figure 3**) but not at baseline or during the recovery phase (**Supplementary Figure 1**). Although high percentages of explained variance in PC1 (72.4%) has been observed, the treatment effect on elemental compositions during water-deficit phase was observed at PC2 with 11.8% explained variance and the variation was mostly driven by CHO and CHON (**Figure 3**).

**Table 1:** Permutational multivariate analysis of variance (PERMANOVA) results of elemental composition for baseline (day 0), water deficit (day 2-11), and recovery phase (day 14-18) data (n=5).

	Factors	df	R^2^	F	*p*-value
Baseline	Treatment	1	0.08	0.72	ns
	Residual	8	0.92		
	Total	9	1		
Water-deficit phase	Treatment	1	0.05	2.6	0.07
	Days	4	0.10	1.37	ns
	Treatment×Days	4	0.09	1.19	ns
	Residual	40	0.76		ns
	Total	49	1		ns
Recovery phase	Treatment	1	0.02	0.58	ns
	Days	2	0.12	1.78	ns
	Treatment×Days	2	0.03	0.46	ns
	Residual	24	0.83		
	Total	29	1		

### 3.3 Distribution of drought-induced metabolites in the water-deficit phase

**Figure 4:**
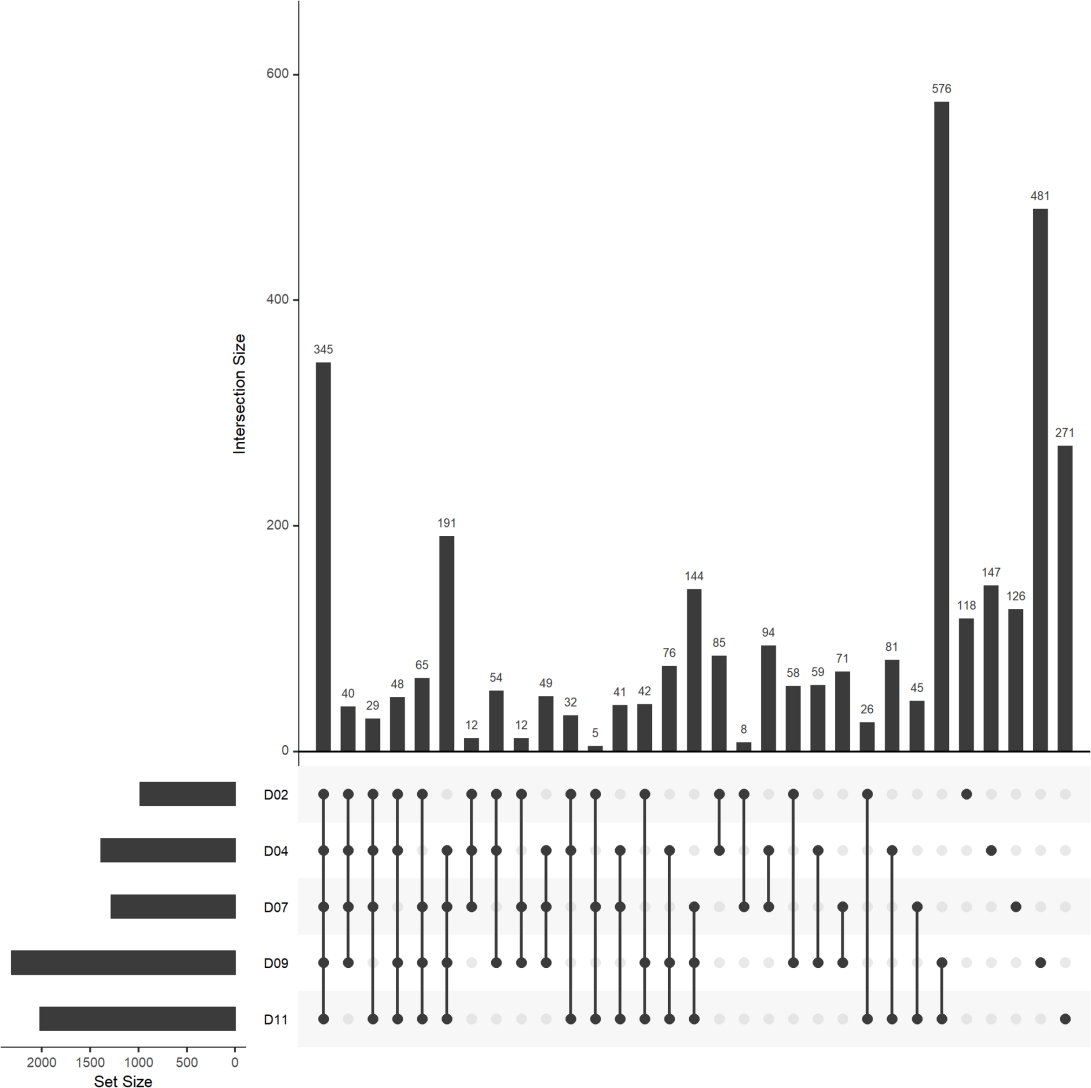
UpSet plot of drought-unique peaks (assigned molecular formula) by each day during water-deficit phase (day 2-11, n=5). UpSet plots visualize set intersections in a matrix layout. The bar plot on the left indicates the total number of unique peaks identified in drought-treated samples (D) at day 2 to 11. The bar chart at the top right and the number on top of each bar indicate the intersection sizes across different days. The connected dots at the bottom right indicate the days used for each intersection.

The unique peaks identified from the drought-treated samples were further visualized using an UpSet plot (**Figure 4**). There were 345 metabolites consistently identified throughout the water-deficit phase. The number of distinct metabolites identified at individual days ranged from 118 to 481 with the highest number identified at day 9 in the drought treatment (**Figure 4**). While looking at the different intersections across the water-deficit phase, there were > 500 unique metabolites identified under severe drought stress (day 9-11) and < 100 metabolites uniquely identified at early drought stress (day 2-4) (**Figure 4**).

### 3.4 Functional characteristics of the drought-induced unique metabolites

KEGG annotation analysis identified a total of 68 pathways and over 17 modules that were induced under water-deficit phase (**Tables 2**, **3**; **Supplementary Tables 2**, **3**). In the drought treatment, 30 out of 68 pathways were consistently observed from day 2 to 11 including flavonoid biosynthesis, carotenoid biosynthesis, phenylpropanoid biosynthesis, and plant hormones biosynthesis (**Table 2**). A trend of increased counts was observed among those consistently induced pathways and modules (**Tables 2**, **3**). The highest counts for drought-induced unique metabolites across most pathways were primarily observed on day 9 (severe drought) followed by a reduction at day 11 (near wilting point) (**Table 2**). Several pathways that might be associated with microorganisms, such as phosphotransferase system (PTS), biofilm formation and quorum sensing, and biosynthesis of several antibiotics were also identified. It is important to note that many assigned molecular formulas were mapped to higher KEGG hierarchies “metabolic pathway” and “biosynthesis of secondary metabolites” without further classification (**Table 2**).

**Table 2:** Potential KEGG pathways with number of drought-induced unique metabolites in the water-deficit phase (day 2-11, n=5).

		Days of treatments						
Group	KEGG pathways	2	4	7	9	11	Comments	References
Unclassified metabolic pathways	Metabolic pathways	125	189	246	408	269		
Unclassified secondary metabolites	Biosynthesis of secondary metabolites	150	208	264	528	326		
	Biosynthesis of various plant secondary metabolites	13	13	19	39	28		
	Biosynthesis of various other secondary metabolites	4	4	4	8	7		
	Biosynthesis of plant secondary metabolites	9	45	39	49	48		
	Biosynthesis of type II polyketide products	16	0	0	36	13		
Phenylpropaniods	Biosynthesis of phenylpropanoids	35	38	29	137	71	Phynelpropanoids are synthesized from phenylalanine and tyrosine. The phenylpropanoid pathway serves as a starting point for production of important metabolites such as flavonoids and stibenoid	Fraser and Chapple, 2011; Winkel-shirley, 2001
	Sesquiterpenoid and triterpenoid biosynthesis	26	26	42	80	29		
	Phenylpropanoid biosynthesis	11	5	11	34	26		
Phenolic compounds	Stilbenoid, diarylheptanoid and gingerol biosynthesis	5	7	8	32	27	Upregulated under drought stress in maize	Wang et al., 2018
Flavonoids	Flavonoid biosynthesis	54	35	29	111	77	Flavonoids are a diverse group of phenolic acids. Flavonoids have been identified in root exudates and induced by drought. Flavonoids are signaling compounds in rhizosphere symbiosis with mycorrhizal fungi and nodulating bacteria	Hassan and Mathesius, 2012; Gargallo-Garriga et al., 2018; Ghatak et al., 2022
	Isoflavonoid biosynthesis	25	15	6	101	35		
	Flavone and flavonol biosynthesis	0	0	0	42	11		
Carotenoid	Carotenoid biosynthesis	23	38	46	52	52	Carotenoid and zeaxanthin as precursors lead to diverse products, including abscisic acid	Wasilewska et al., 2008
Fatty acid	Alpha-linolenic acid metabolism	15	26	19	40	28	Alpha-linolenic acid is unsaturated fatty acid and the precursor of jasmonic acid	Wasternack and Hause, 2013
	Linoleic acid metabolism	8	13	20	50	33	Unsaturated fatty acids serve as ingredient of extracellular barrier constituents such as cutin and suberin; and stress signaling molecules	He and Ding, 2020
	Biosynthesis of unsaturated fatty acids	11	24	22	47	26		
	Fatty acid biosynthesis	10	18	12	18	18		
	Fatty acid degradation	1	1	1	1	1		
	Fatty acid elongation	1	1	1	1	1		
	Fatty acid metabolism	1	1	1	1	1		
Plant hormone	Biosynthesis of plant hormones	0	25	25	28	28	Multiple phytohormones have been reported to be associated with drought response, including but not limited to abscisic acid and jasmonic acid	
	Plant hormone signal transduction	0	25	25	25	25		Chhaya et al., 2021; El Sabagh et al., 2022
	Insect hormone biosynthesis	1	2	15	45	6		
	Steroid hormone biosynthesis	1	1	5	5	5		
Other Terpenoids and Steroids	Biosynthesis of terpenoids and steroids	15	38	38	41	41	Specialized terpenoids might play an important role in response to abiotic and biotic stresses, as signals and rewards to mycorrhizal fungi	Pichersky and Raguso, 2018
	Ubiquinone and other terpenoid-quinone biosynthesis	2	2	2	34	17		
	Diterpenoid biosynthesis	0	0	18	31	0		
	Monoterpenoid biosynthesis	2	3	1	12	14		
	Terpenoid backbone biosynthesis	0	0	0	19	0		
	Biosynthesis of alkaloids derived from terpenoid and polyketide	0	0	0	2	2		
	Steroid degradation	0	1	0	1	0		
Amino acid	Tyrosine metabolism	1	1	1	12	1	Phenylalanine and tyrosine are the precursors of phenylpropanoids	Winkel-shirley, 2001; Fraser and Chapple, 2011
	Phenylalanine, tyrosine, and tryptophan biosynthesis	3	0	3	6	6		
	Biosynthesis of amino acids	0	0	0	7	3		
	Lysine biosynthesis	0	0	0	4	0		
Cell death pathway	Ferroptosis	0	0	0	17	0	A programmed cell death process that has been linked to heat stress	Distéfano et al., 2017; Distéfano et al., 2022
Antibiotics	Biosynthesis of various antibiotics	48	73	94	105	76	Produced by some plant endophytes such as *Streptomyces* and *Pseudomonas*	Steffensky et al., 1998; Laursen and Nielsen, 2004; Li et al., 2015; Berg et al., 2020
	Novobiocin biosynthesis	0	1	1	12	12		
	Biosynthesis of enediyne antibiotics	0	0	3	8	6		
	Phenazine biosynthesis	0	0	0	3	3		
Microorganisms related	Phosphotransferase system (PTS)	0	41	38	44	44		
	Bacterial chemotaxis	0	38	38	38	38		
	Naphthalene degradation	0	2	2	20	10		
	Biofilm formation - Vibrio cholerae	0	2	2	2	0		
	Quorum sensing	0	2	2	2	0		
	Aflatoxin biosynthesis	0	0	0	11	1		
	Microbial metabolism in diverse environments	9	7	0	37	12		
Alkaloids	Biosynthesis of various alkaloids	0	0	4	10	7		
	Tropane, piperidine and pyridine alkaloid biosynthesis	3	3	3	3	3		
	Biosynthesis of alkaloids derived from shikimate pathway	0	0	0	3	3		
Aromatic compound degradation	Degradation of aromatic compounds	3	1	0	16	1		
	Polycyclic aromatic hydrocarbon degradation	0	0	0	3	3		
Glycolysis	Glycolysis/Gluconeogenesis	0	3	0	6	6		
	Glucosinolate biosynthesis	0	0	0	4	4		
	Pyruvate metabolism	0	0	0	4	0		
Signal transduction	GABA-A receptor agonists/antagonists	2	2	2	2	2	GABA is a regulator of malate efflux anion channels. Malate is one of the critical compounds that plants exude to mobilize inorganic P in soil	Ramesh et al., 2015; Krishnapriya and Pandey, 2016
	cAMP signaling pathway	0	0	17	0	0		
	ABC transporters	0	38	38	38	38	Associated with transport of the phytohormones and root exudation	Badri et al., 2009; Kang et al., 2011
	Two-component system	2	6	8	8	6		
Other	Cutin, suberine and wax biosynthesis	24	37	39	58	57		
	Biosynthesis of 12-, 14- and 16-membered macrolides	3	4	21	6	4		
	2-Oxocarboxylic acid metabolism	3	0	3	11	7		
	Biosynthesis of cofactors	0	0	0	19	15		
	Propanoate metabolism	0	0	0	4	0		
	Biosynthesis of siderophore group on ribosomal peptides	0	0	0	3	3		
	Folate biosynthesis	0	0	0	3	3		
	Metabolism of xenobiotics by cytochrome P450	0	0	0	1	1		
	Xylene degradation	0	0	0	1	0		

The number indicates the total count of assigned molecular formula by day that are unique to drought treatment. Pathways with similar properties were grouped.

**Table 3:** Potential KEGG modules with number of drought-induced unique metabolites in the water-deficit phase (day 2-11, n=5).

	Days of treatment					
KEGG module	2	4	7	9	11	Total
Pentalenolactone biosynthesis, farnesyl-PP => pentalenolactone	4	5	6	6	5	26
Jasmonic acid biosynthesis	2	3	2	3	2	12
Abscisic acid biosynthesis	1	2	2	2	2	9
Menaquinone biosynthesis	0	0	0	6	6	12
Erythromycin biosynthesis	2	2	2	2	2	10
Pterocarpan biosynthesis, daidzein => medicarpin	2	2	1	4	0	9
Oleandomycin biosynthesis	1	2	2	2	2	9
Flavanone biosynthesis	1	1	1	3	3	9
Isoflavone biosynthesis	0	0	0	3	3	6
Phylloquinone biosynthesis	0	0	0	4	4	8
Bile acid biosynthesis	0	0	2	2	3	7
Dihydrokalafungin biosynthesis	1	0	0	4	2	7
Monolignol biosynthesis	1	0	0	4	2	7
Futalosine pathway	0	0	0	3	3	6
Kedarcidin 2-hydroxynaphthoate moiety biosynthesis	0	0	2	2	2	6
Lovastatin biosynthesis	1	1	1	2	1	6
Modified futalosine pathway	0	0	0	3	3	6

The number indicates the total count of assigned molecular formula by day that are unique to drought treatment. Listed KEGG modules include only unique formula with total counts greater than 5 throughout entire water-deficit phase for each KEGG module are listed.

### 3.5 Higher biochemical transformations were identified under water-deficit plants

A total of 99 potential biochemical transformations were identified in this study (**Table 4**; **Supplementary Table 4**). Over 97% of the identified transformations had the highest number of counts in the drought-treated exudates at day 9, including methylation (-H), oxidation/hydroxylation (-H), hydrogenation/dehydrogenation, ethyl addition (-H_2_O), etc. (**Table 4**; **Supplementary Table 4**). The increase of transformation along the progressive drought stress showed a similar trend that was identified in the mass distribution and KEGG annotation results.

**Table 4:** Potential biochemical transformations identified throughout water-deficit phase (day 2-11, n=5) as the total counts of assigned molecular formula by day.

	Days of treatment											
	0		2		4		7		9		11	
Transformation	Control	Drought	Control	Drought	Control	Drought	Control	Drought	Control	Drought	Control	Drought
Methylation (-H)	321	165	373	257	896	467	860	548	870	1465	1023	966
Ethyl addition (-H_2_O)	240	136	288	211	803	393	800	502	744	1233	913	808
Oxidation/hydroxylation (-H)	349	138	276	232	679	300	613	417	711	1338	799	799
Hydrogenation/dehydrogenation	274	62	197	140	505	199	406	301	610	1166	675	721
Formic acid (-H_2_O)	179	31	134	98	453	179	372	232	492	944	542	551
Condensation/hydration/dehydration	210	51	159	118	378	153	308	233	422	906	481	522
C2H2	159	39	112	86	393	135	335	201	454	893	533	531
Acetylation (-H_2_O)	149	28	119	91	418	161	350	210	443	895	527	500
Nitro reduction (-O_2_)	136	31	134	95	441	176	362	237	424	797	456	452
Carboxylation	123	22	113	69	418	158	343	197	379	814	449	460
Glyoxylate (-H_2_O)	93	11	68	48	254	98	205	123	285	631	315	339
Malonyl group (-H_2_O)	62	4	52	32	240	82	198	109	251	536	301	305
Erythrose (-H_2_O)	68	11	43	31	236	79	215	108	262	510	279	303
Acetotacetate (-H_2_O)	65	9	37	26	204	68	162	84	250	522	270	297
D-ribose (-H_2_O) (ribosylation)	47	9	18	16	134	43	112	64	187	362	184	190
Glucoside conjugation (-H)	59	6	24	17	92	35	62	49	145	300	155	158
Secondary amine	62	10	39	13	57	27	35	46	105	288	113	181
Thiourea to urea (-S)	24	13	38	27	65	39	75	50	66	204	87	127
Glycine conjugation (-OH)	29	2	27	11	56	25	29	26	81	256	98	141
Oxidative deamination to ketone (-H_3_N)	34	7	33	21	39	17	28	40	71	198	77	125

Only the top 20 abundance reactions were listed. All samples and assigned molecular formula were included in this analysis.

## 4 Discussion

The use of aeroponics systems allows for non-destructive root exudate collection under continuous treatments, eliminating the interference from soil particle absorption, root damage, and microbial decomposition (Oburger and Jones, 2018). In aeroponics, the abundance of root hairs and the root structure may differ compared to plants grown in soil (Eldridge et al., 2020). However, it has been reported that in cassava (*Manihot esculenta*), the genotype response to plant hormones remains consistent between aeroponic and field experiment (Selvaraj et al., 2019). This suggests that the plant’s response in aeroponics can be used to predict its response in the field. Nevertheless, further investigations are necessary to understand the differences in root exudate profiles in various cultivation environments. To the best of our understanding, this was the first time that drought-induced metabolome in cotton root exudates have been characterized, mapped to KEGG databases, and reported.

Previous work has shown that the concentration of primary metabolites in plant roots, such as methanol, glucose, small aminos acids, and other low molecular weight compounds changes under drought (Honeker et al., 2022). Since we focused on compounds with the m/z range of 200 to 900, we were not able to capture most of the low molecular weight compounds, which is also an important component of root exudates. Our research specifically focused on plant secondary metabolites in response to drought. The KEGG and biochemical transformation analysis suggested that changes to the drought-induced metabolome occur in a targetable groups of metabolic pathways (Wang et al., 2019), and the reactions take place intensively when plant experience severe drought. Although PERMANOVA was not able to detect differences in elemental composition between treatments and days of treatment during either the water-deficit or recovery phase (**Table 1**). This lead to the possibility that the plant alters the structure of metabolites without altering their molecular formula, which are unable to be captured by FT-ICR MS technology (Wang et al., 2019). The high proportion of shared metabolites between control and drought-treated plants (**Figure 2D**) might be associated with plant development, potentially explained why changes in elemental composition induced by drought were not able to be detected *via* PERMANOVA analysis. It has been shown that root exudation profile has larger differences between plant species; however, those differences are not only interspecific, but also intraspecific among individuals, and change over time in response to abiotic stresses (Gargallo-Garriga et al., 2018; Preece and Peñuelas, 2020). Therefore, it was not surprising that unique metabolites at baseline were identified between drought-stressed and control samples (**Figure 2**). To account for variation at baseline, those unique metabolites at baseline were excluded from the KEGG annotation analysis.

### 4.1 Roles of flavonoid compounds in root exudates under drought

During water-deficit phase, > 100 assigned molecular formula in drought-induced root exudates were mapped to pathways associated with the biosynthesis of flavonoids, including isoflavonoid, flavone, and flavonol (**Table 2**; **Supplementary Table 2**). Flavonoids are a group of plant-derived secondary metabolites that consist of > 10,000 compounds (Winkel-shirley, 2001; Sugiyama and Yazaki, 2014). The biosynthesis of flavonoids starts with phenylalanine and malonyl-CoA as the direct precursors. Then it undergoes different chemical reactions, such as hydroxylation, acylation, methylation, malonylation, and prenylation, which results in nine major subgroups (e.g., isoflavonoids, flavones, flavonols, flavandiols, and condensed tannins) having diverse structures and functions (Winkel-shirley, 2001; Sugiyama and Yazaki, 2014). Therefore, it was not surprising that we also observed the pathways associated with the biosynthesis of phenylalanine, tyrosine, and tryptophan; biosynthesis of phenylpropanoids; and KEGG modules involved with biosynthesis of malonyl-CoA under drought conditions (**Table 2**; **Supplementary Table 3**). In addition, a large number of methylation and malonylation transformations were also identified in drought-treated samples (**Table 4**; **Supplementary Table 4**).

Under drought conditions, a greater concentration of flavonoids have been identified in root exudates in holm oak (Gargallo-Garriga et al., 2018) and pearl millet (*Pennisetum glaucum*; Ghatak et al., 2022). Flavonoids have been reported as an important player in drought tolerance by directly functioning as antioxidants or serving as signaling molecules for the symbiosis of plants and microbes (Hassan and Mathesius, 2012). In our study, several masses were mapped to the KEGG module related to “daidzein → medicarpin” reactions (**Table 3**; **Supplementary Table 3**). Daidzein is an isoflavone compound reported to be secreted by soybean roots, as signaling molecules mediating communication between plants and nitrogen-fixing bacteria (Sugiyama et al., 2007; Okutani et al., 2020). It is unclear if cotton also uses similar strategies to attract nitrogen-fixing bacteria. However, several nitrogen-uptake-related bacteria, such as *Mesorhizobium*, *Sinorhizobium*, and *Rhizobium*, have been identified in the cotton rhizosphere (Qiao et al., 2017), which provide potential directions for future studies. Further investigations are required to characterize and quantify the flavonoid compounds in cotton root exudates and the associated microbes.

### 4.2 Plant hormones in root exudates

It has been well documented that multiple ubiquitous plant hormones, including abscisic acid (ABA) and jasmonic acid (JA), are coordinated in response to drought stress (Wang et al., 2021; El Sabagh et al., 2022). In this study, drought treatment induced the pathways and modules involved in the biosynthesis of ABA and JA, as well as their associated precursors, carotenoids (Wasilewska et al., 2008) and alpha-linolenic acid (Wasternack and Hause, 2013) (**Tables 2**, **3**). In a previous study, we were able to detect ABA in collected root exudates using GC-MS, although the differences between drought and control treatment were only detected at certain days during water deficit phase (Lin et al., 2022, preprint). We also identified the pathway associated with ATP-binding cassette (ABC) transporters induced under drought, which have been reported to transport phytohormones and root exudation (Badri and Vivanco, 2009; Kang et al., 2011). Because our root exudation collection method was non-destructive compared to other sampling approaches (Oburger and Jones, 2018), we assume that cotton undergoes active transport of those plant hormones into the rhizosphere in order to regulate hormone concentrations inside the root tissues in response to drought, as previously reported in upland rice (*Oryzae sativa*) (Shi et al., 2014). However, it would be necessary to identify the associated transporters to support this assumption and further understand the mechanisms behind this finding.

### 4.3 Antibiotics identified in root exudates potentially associated with root endophytes

Interestingly, pathways associated with the biosynthesis of antibiotics (e.g., novobiocin, enediyne antibiotics, and phenazine) were identified under drought, even in soil-less environments. Those antibiotics have been reported to be produced by a variety of saprophytic or endophytic microorganisms (Steffensky et al., 1998; Laursen and Nielsen, 2004; Chen et al., 2010; Li et al., 2015). *Streptomyces*, which includes both endophytic and saprophytic species, has been known to produce novobiocin and enediyne antibiotics (Steffensky et al., 1998; Chen et al., 2010). Phenazine is also a common antibiotic that can be isolated from *Streptomyces* and *Pseudomonas* (Laursen and Nielsen, 2004; Li et al., 2015) that is known to be involved in biofilm formation, which is important process in drought tolerance in plant roots (Mahmoudi et al., 2019). Our growth chamber experiment was not performed in a sterile environment, so by using a soilless aeroponic setup, is it likely that those antibiotics might be produced by host endophytes. Indeed, studies have shown that *Streptomyces* was enriched in the root under drought and expected to be associated with improving plant fitness (Xu and Coleman-Derr, 2019). *Pseudomonas* also has been reported as one of the dominant endophytic organisms in cotton roots (Shi et al., 2020). Our current experiment design is not able to differentiate the metabolites that were released by plants or endophytes; however, incorporating isotope labeling techniques could further reveal the mechanisms between plants and microbes under drought.

### 4.4 Major shift of metabolomic profile during severe drought, but not wilting point

A major shift in the metabolomic profile occurred in our study under severe drought prior to the plant reaching wilting point. Our findings provide temporal responses to progressive drought with activation of various metabolic pathways, which provide important information on relevant sampling time points to further characterize and validate the drought-response mechanisms in cotton root exudates. The transition from severe drought stress to wilting point (day 9 to 11) may be related to cell death, giving insight into the complicated senescence of plants and how root exudates respond to plant death, which has limited attention in recent studies (Prudence et al., 2021). One pathway, ferroptosis, associated with cell death, was identified exclusively on day 9 (**Table 2**). Ferroptosis is an iron-dependent and highly regulated cell death process (Riyazuddin and Gupta, 2021; Distéfano et al., 2022). In *Arabidopsis thaliana*, this programmed cell death process was induced by heat stress and was mainly dependent on reactive oxygen species (ROS)-mediated lipid peroxidation (Distéfano et al., 2017). Currently, there are few studies on this newly discovered pathway and no reports indicating whether drought stress will induce this pathway. However, many abiotic stresses, including drought, also induce the accumulation of ROS in plant tissues (Lee and Park, 2012). Shared exudation patterns among heat, drought, and the combination of both stresses have been reported in maize (Tiziani et al., 2022), indicating there are shared responses when plants encounter these types of stresses. The small amount (only 17) of assigned molecular formula that were mapped to the ferroptosis cell death pathway, which were only identified during severe drought (day 9) in our study, suggests that the plant might release “final warning stress signals” prior to wilting point, but it also suggests there might be minor confounding responses between drought and cell death at this collection timepoint.

## 5 Conclusions

Our study suggested that cotton released unique metabolites in root exudates depending on drought severity. With the use of FT-ICR MS, we were able to characterize all drought-induced metabolites with a m/z range of 200 to 900. The drought-induced metabolome occurred in a small and targetable group of metabolic pathways. To further isolate the specific compounds, targeting the plant under severe drought instead of the wilting point is recommended. By utilizing a non-destructive sampling approach, we were able to capture the temporal changes of exudation profiles of the same plant without the interference of soil particles. Our findings advance the fundamental characteristics of root exudates and plant drought-tolerance mechanisms belowground. Future research should focus on: (1) characterizing the unclassified metabolites, particularly secondary metabolites; (2) utilizing targeted metabolomic approaches (e.g., LC-MS, GC-MS, or HPLC) to quantify the concentration of several drought-induced candidate groups and confirm metabolite annotations, such as flavonoids, to further verified their importance to the plant drought response; and (3) incorporate the results in greenhouse or field trials to further understand the consequence of drought on rhizosphere microbiome and plant performance.

## Data availability statement

The original contributions presented in the study are included in the article/**Supplementary Material**. Further inquiries can be directed to the corresponding author.

## Author contributions

H-AL contributed to data curation, formal analysis, investigation, methodology, visualization, writing – original draft, writing – review & editing. HRC contributed to data curation, formal analysis, investigation, methodology, writing – review & editing. JAH contributed to conceptualization, funding acquisition, investigation, methodology, resources, supervision, writing – review & editing. MMT contributed to SPE extraction, FT-ICR MS analysis and data curation, writing – review & editing. EMN contributed to methodology, writing – review & editing. SA-B contributed to resources, writing – review & editing. SH contributed to germplasm resources, writing – review & editing. APS contributed to conceptualization, funding acquisition, investigation, methodology, project administration, resources, supervision, writing – review & editing. All authors contributed to the article and approved the submitted version.
