# Supplementary figures and images for: Progressive drought alters the root exudate metabolome and differentially activates metabolic pathways in cotton (Gossypium hirsutum)
Source: Front Plant Sci. 2023 Aug 30;14:1244591. doi: 10.3389/fpls.2023.1244591 (PMC10499043; doi:10.3389/fpls.2023.1244591)

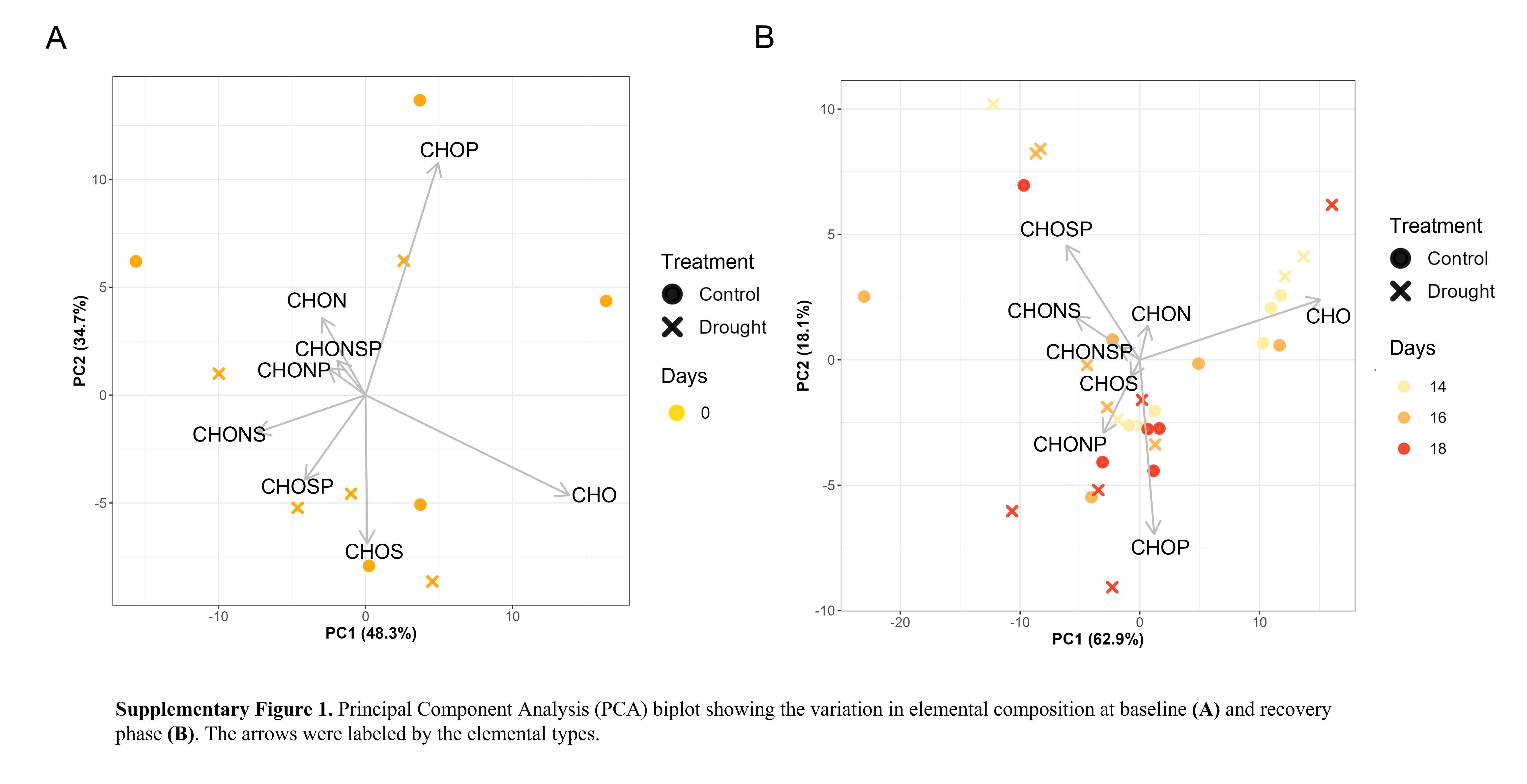

Supplement: Supplementary file 1 [file Image_1.tif]
